# Synergistic antitumour activity of RAF265 and ZSTK474 on human TT medullary thyroid cancer cells

**DOI:** 10.1111/jcmm.12612

**Published:** 2015-06-17

**Authors:** Loris Bertazza, Susi Barollo, Claudia Maria Radu, Elisabetta Cavedon, Paolo Simioni, Diego Faggian, Mario Plebani, Maria Rosa Pelizzo, Beatrice Rubin, Marco Boscaro, Raffaele Pezzani, Caterina Mian

**Affiliations:** a Endocrinology Unit, Department of Medicine, University of PaduaPadua, Italy; b 5^th^ Chair of Internal Medicine, Department of Medicine, University of PaduaPadua, Italy; c Department of Laboratory Medicine, Padua University HospitalPadua, Italy; d Department of Surgical, Oncological and Gastroenterological Sciences (DiSCOG), Surgery Unit, University of PaduaPadua, Italy

**Keywords:** RAF265, SB590885, ZSTK474, BRAF, RET, medullary thyroid cancer

## Abstract

Medullary thyroid cancer (MTC) is an aggressive malignancy responsible for up to 14% of all thyroid cancer-related deaths. It is characterized by point mutations in the rearranged during transfection (*RET*) proto-oncogene. The activated RET kinase is known to signal *via* extracellular signal regulated kinase (ERK) and phosphoinositide 3-kinase (PI3K), leading to enhanced proliferation and resistance to apoptosis. In the present work, we have investigated the effect of two serine/threonine-protein kinase B-Raf (BRAF) inhibitors (RAF265 and SB590885), and a PI3K inhibitor (ZSTK474), on RET-mediated signalling and proliferation in a MTC cell line (TT cells) harbouring the *RETC634W* activating mutation. The effects of the inhibitors on VEGFR2, PI3K/Akt and mitogen-activated protein kinases signalling pathways, cell cycle, apoptosis and calcitonin production were also investigated. Only the RAF265+ ZSTK474 combination synergistically reduced the viability of treated cells. We observed a strong decrease in phosphorylated VEGFR2 for RAF265+ ZSTK474 and a signal reduction in activated Akt for ZSTK474. The activated ERK signal also decreased after RAF265 and RAF265+ ZSTK474 treatments. Alone and in combination with ZSTK474, RAF265 induced a sustained increase in necrosis. Only RAF265, alone and combined with ZSTK474, prompted a significant drop in calcitonin production. Combination therapy using RAF265 and ZSTK47 proved effective in MTC, demonstrating a cytotoxic effect. As the two inhibitors have been successfully tested individually in clinical trials on other human cancers, our preclinical data support the feasibility of their combined use in aggressive MTC.

## Introduction

Thyroid cancer is the most common malignant tumour of the endocrine system and accounts for approximately 1% of all newly diagnosed cancer cases in the United States. 1. A small proportion of thyroid malignances are cases of parafollicular or C-cell-derived medullary thyroid cancer (MTC) b, which is much more aggressive than the well-differentiated papillary and follicular malignancy and is reportedly responsible for up to 14% of all deaths related to thyroid cancer 3. More than 50% of patients with MTC have cervical lymph node metastases at the time of their diagnosis, and up to 5% have distant metastases. Medullary thyroid cancer is usually unresponsive to conventional cytotoxic chemotherapy and radiation therapy 4, and it is prone to metastasize early, so the recommended initial treatment usually includes radical excision of the thyroid together with central neck lymph node dissection 5.

The main secretory product of the parafollicular cells in MTC is calcitonin (CT), which is therefore the reference tumour marker for this neoplasm 6.

**Figure 1:**
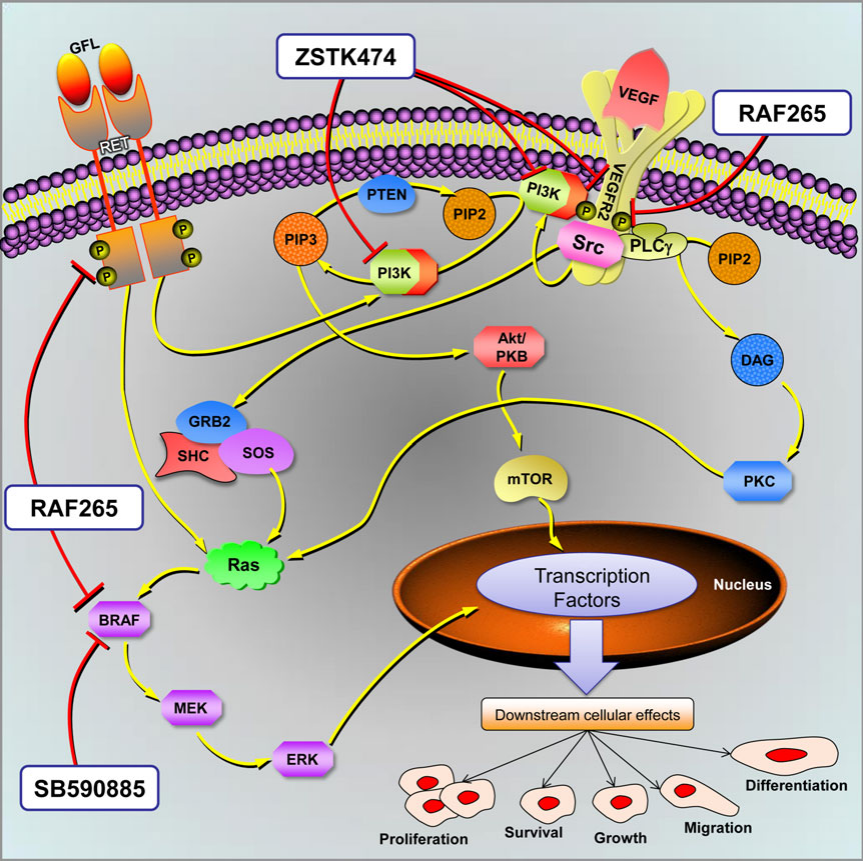
Proposed model for multiple-target inhibitory action in TT cells. This figure schematically shows the tyrosine kinase receptors, such as RET, VEGFR2, and some of their downstream effectors. The two most important oncogenic signalling pathways are PI3K/Akt/mTOR and Ras/Raf/MEK/ERK. Activation of these pathways may transduce different transcription factors and induce tumour cell proliferation, survival and migration, with subsequent tumour growth, lymphangiogenesis/angiogenesis, and metastasis. RAF265, SB590885 and ZSTK474 kinase inhibitors and their targets are shown too. Pathway map modified from SABiosciences.

The rearranged during transfection (*RET*) proto-oncogene located on chromosome 10q11.2 codes for a transmembrane receptor (RET) that has tyrosine kinase activity. Point mutations in *RET* are identified in about 98% of cases of familial MTC, and in 30–50% of sporadic cases 7. The *RET* gene encodes the signalling subunit of a receptor complex for ligands of the glial-derived neurotrophic factor family 8, which in turn binds to a family of glial cell line-derived neurotrophic factor (GDNF) family receptor α (GFRα) co-receptors, consisting of four glycosylphosphatidylinositol-anchored proteins, GFRα1-4, that form a complex with RET tyrosine kinase. The function of RET has been extensively studied *in vivo*, and in various cell types, *in vitro* 9,10. The activated RET kinase transmits mitogenic, survival and motogenic signals. Two major signalling cascades, namely rat sarcoma (Ras) and phosphatidylinositol 3-kinase (PI3K), are triggered by RET (Fig.1) 11. In turn, Ras and PI3K contribute to the activation of many signalling effectors. The Ras/Raf/MEK/ERK cascade is the best characterized Ras-effector pathway. There are three Raf serine/threonine kinases (ARAF, BRAF and CRAF) that activate the MEK-extracellular signal regulated kinase (ERK) kinase cascade. ERK, in turn, stimulates gene transcription by directly phosphorylating transcription factors or by targeting intracellular kinases 12. Class I PI3K is constituted by a regulatory and a catalytic subunit. Upon recruitment to the plasma membrane by activated RET, PI3K phosphorylates phosphatidylinositol-4, 5-bisphosphate (PIP2) to generate phosphatidylinositol-3, 4, 5-triphosphate (PIP3; Fig.1). PIP3, in turn, activates downstream molecules such as Akt (also known as PKB) serine/threonine kinase, which stimulates the serine/threonine kinase mammalian target of rapamycin (mTOR). The PI3K/Akt/mTOR cascade is important in MTC tumorigenesis because of its ability to promote growth and proliferation and to prevent cell death 13. Switching off Ras/Raf/MEK/ERK and PI3K/Akt/mTOR signalling pathways may be of therapeutic benefit in patients with oncological disease characterized by activation of these 2 pathways. In our previous work, we tested three new drugs, two serine/threonine-protein kinase B-Raf (BRAF) inhibitors (RAF265 and SB590885) and a PI3K inhibitor (ZSTK474), on differentiated thyroid cancer cell lines carrying the *BRAF V600E* mutation, discovering that the combination of drugs profoundly affected proliferation *via* the mitogen-activated protein kinases (MAPK) and PI3K/Akt signalling pathways 14. Given the crosstalk between these pathways, we examined the inhibitory effects of these compounds, alone or in combination, on an aggressive TT MTC cell line harbouring the *RET C634W* activating mutation 15.

## Materials and methods

### Cell culture

The TT cell line (MTC, human) was obtained from the European Collection of Cell Cultures (Sigma-Aldrich, Milano, Italy) and cultured in RPMI 1640 (Gibco - Life Technologies, Carlsbad, CA, USA) supplemented with 10% foetal bovine serum (FBS; Gibco), L-glutamine (2 mM) and penicillin-streptomycin (100 IU/ml–100 μg/ml respectively). Adherent monolayer cells were maintained in T-75 culture flasks and incubated at 37°C with 5% CO_2_ until they achieved 85% confluence. Cells were detached using 0.25% trypsin-EDTA (Sigma-Aldrich) and plated in T-75 flasks at a density of 2 × 10^6^ cells.

RAF265 was kindly provided by Novartis International (Basel, Switzerland), SB590885 and ZSTK474 were purchased from Selleck Chemicals (Houston, TX, USA). The powders were dissolved in a 10 mM stock solution in dimethyl sulfoxide (DMSO), following the manufacturer’s instructions.

### MTT cell viability assay and drug synergism

The TT cells were plated on 96-well tissue culture microtiter plates at a density of 1 × 10^4^ cells/well and treated with RAF265, SB590885 and ZSTK474 at different concentrations (range 0.01–100 μM). MTT cell viability (Sigma-Aldrich) was tested after 72 hrs of treatment, as described elsewhere 14. For each drug, we measured the Inhibitory Concentration 50 (IC50, defined as 50% of the inhibitory effect on cell viability). All experiments were performed in quadruplicate and repeated three times.

The combination index (CI) values were calculated using the CompuSyn 3.0.1 program (Ting-Chao Chou and Nick Martin). Based on the dose–response curves, using the MTT assay for cells treated with inhibitors, alone or in combination at a constant ratio, a series of CI values were generated over a range of levels of growth inhibition (GI) from 5% to 95% of the fraction affected. The values at 50% GI are presented for the RAF265+ ZSTK474 and SB590885+ ZSTK474 combinations. Synergism, additive effect, and antagonism are defined as CI < 1, CI = 1, and CI > 1 respectively.

### Trypan blue cell viability assay

The cytotoxic effects of RAF265, ZSTK474 ad SB590885, alone and in combination were confirmed by the Trypan blue dye exclusion method. The assay was performed at 72 hrs after treatment, using the single IC50 dose determined by MTT assay. At the end of treatment, cells were collected by trypsinization, centrifuged and the cell pellet was resuspended in 1 ml of PBS. Next, 10 μl of the resulting cell suspension was admixed with 10 μl of Trypan blue (0.4% in PBS). The numbers of non-stained viable cells (NSt cells) and stained dead cells (St cells) were counted using a hemocytometer. Cell viability was then calculated by the following formula:

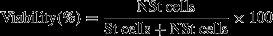


Experiments were performed in triplicated and repeated three times. The results were interpreted as the ratio of viable cells after drug treatments to that of the untreated control.

### Western blot analysis

The TT cells were treated for 4 hrs in 60 mm cell culture dishes with the IC50 concentrations of the drugs, alone and in combination. Proteins were extracted and centrifuged, and the supernatant was collected. Total proteins were quantified, separated by SDS/PAGE, electro-blotted onto nitrocellulose membranes and saturated in 5% fat-free dried milk. Membranes were incubated overnight with primary antibodies and then incubated with secondary ones (Sigma-Aldrich). Immunoreactivity was detected with Euroclone ECL long-lasting substrate (Euroclone, Milano, Italy). The primary antibodies were anti-Erk1/2, anti-phospho-Erk1/2 (Thr202/Tyr204), anti-Akt, anti-phospho-Akt (Ser473), anti-VEGFR2, anti-phospho-VEGFR2 (Tyr1175), all (1:1000) from Cell Signaling (Danvers, MA, USA), and anti-β-actin (1:5000) from Sigma-Aldrich. Films were scanned and the band intensity was quantified with ImageJ software 1.44p. All experiments were performed in triplicate.

### Calcitonin measurement

Calcitonin transcriptional levels were determined by quantitative real-time PCR (qrtPCR) using the specific TaqMan assay Hs01100741_m1, and the corresponding quantification with 2^−ΔΔCt^ method. TT cells were treated for 24, 48 and 72 hrs with the drugs, alone and in combination. The method was validated by verifying the amplification efficiency of the target gene and the beta-actin housekeeping gene (Hs99999903_m1). The qrtPCR was performed with TaqMan assays (Life Technologies, Monza, Italy) in an ABI PRISM 7900 HT Sequence Detection system. The experiment was performed in triplicate.

Calcitonin secretion was quantified in the TT cells in the conditioned medium after treatment with the drugs (alone and in combination). Briefly, cells were plated in 60 mm tissue culture dishes and treated for 24, 48 and 72 hrs. At the end of each time interval, the conditioned medium was collected and the CT levels were quantified using LIAISON® Calcitonin_II-Gen (DiaSorin Inc., Stillwater, MN, USA), an analytical method that employs a chemiluminescent immunoassay 16. The experiment was performed in triplicate.

### Cell cycle analysis

Cells were plated in 100 mm cell culture dishes at a density of 2 × 10^6^ cells and treated with the drugs, alone or in combination, for 24 hrs (data not shown) and 72 hrs, as described elsewhere 14. Cell cycle analysis was performed in triplicate using the Cytomics FC500 and the data were analysed with the CXP software (Beckman Coulter, Fullerton, CA, USA).

### Apoptosis

Apoptosis was tested after 48 hrs of treatment with the Annexin V-FITC kit (BD Biosciences, San Diego, CA, USA), as described previously 14. Briefly, all cells were plated in 25 cm^2^ flasks at a density of 1 × 10^6^ cells/well for 2 days, then maintained overnight at 0.1% FBS. The next day, the cells were treated with the drugs, alone or in combination, for 48 hrs, then trypsinized and harvested by centrifugation. The cells were then stained with Annexin V-FITC/propidium iodide according to the manufacturer’s instructions. All analyses were performed in triplicate using the Cytomics FC500 (Beckman Coulter).

### Statistical analysis

Nonlinear regression and sigmoidal dose–response curves were used to calculate the IC50 values. Normality tests were applied (the Kolmogorov–Smirnov, D’Agostino and Pearson omnibus normality, and Shapiro–Wilk tests), then group comparisons were drawn using either one-way anova with Bonferroni’s post-hoc test or the unpaired *t*-test. Significance was assumed for a *P* < 0.05. All analyses were run using GraphPad Prism rel. 5.03 for Windows (GraphPad Software, San Diego, CA, USA).

## Results

### IC50 values and synergistic effects of RAF265, SB590885 and ZSTK474 in the TT cell line

**Figure 2:**
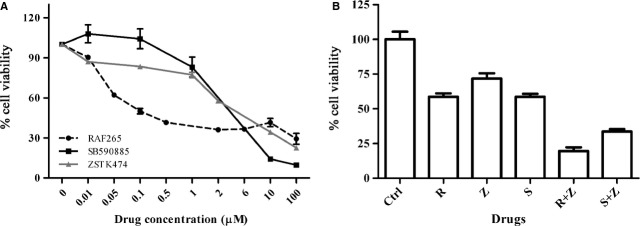
Cell viability of TT cells. (A) Cell viability was estimated by MTT assay after 72 hrs of treatment with RAF265, SB590885 and ZSTK474. Each analysis was performed with 4 replicates and repeated in 3 separate experiments. (B) Effects on cell viability by Trypan blue dye exclusion method after 72 hrs of treatment with RAF265 (R), ZSTK474 (Z) and SB590885 (S), alone and in combination (R+Z and S+Z) using the single IC50 doses determined by MTT assay. Each analysis was performed with 3 replicates and repeated in 3 separate experiments.

We examined the *in vitro* effects of RAF265, SB590885 and ZSTK474 on TT cell viability. The IC50 values were measured 72 hrs after treatment (Table1). RAF265 was the most effective compound, showing an IC50 = 0.09 μM, while SB590885 showed an IC50 = 3.36 μM, and ZSTK474 an IC50 = 0.59 μM (Fig.2A).

**Table 1:** IC50 and CI values for TT cells treated with the RAF265, ZSTK474 and SB590885 inhibitors

Cell line	Genotype	IC50 (μM)			CI	
		RAF265	ZSTK474	SB590885	RAF265+ ZSTK474	SB590885+ ZSTK474
TT	RET^C634W^	0.09	0.59	3.36	0.22	1.03

IC50: Inhibitory Concentration 50; CI: combination index.

**Figure 3:**
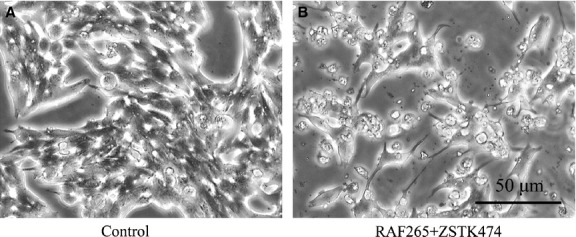
Changes in cell morphology following drug treatments. Effect of the drugs on the morphology of cultured human TT cells after 72 hrs of treatment. (A) No treatment; (B) RAF265 1 μM + ZSTK474 1 μM.

A drug synergy analysis was performed to see whether simultaneously suppressing the MAPK and PI3K/Akt pathways could have inhibitory effects on TT cell viability. Using a wide range of doses, we observed a median CI = 0.22 for the combination RAF265+ ZSTK474, and CI = 1.03 for SB590885+ ZSTK474 (Table1). The effects on cell viability using the IC50 doses, alone and in combination determined by the MTT assay were confirmed by the Trypan blue assay. After 72 hrs, cells treated with RAF265 and SB590885 alone showed a viability of 58% and 59%, respectively, while the PI3K inhibitor ZSTK474 alone caused a decrease in vitality of 72%, compared to control. The synergistic combination RAF265+ ZSTK474 showed a viability reduction of 20%, while SB590885+ ZSTK474 combination showed a viability reduction of 34%, similarly to the additive effect of the two drugs (Fig.2B). The effects of the synergic treatments were also appreciable on the cell’s morphology (Fig.3).

### Effects of the drugs on signalling pathways in the TT cell line

**Figure 4:**
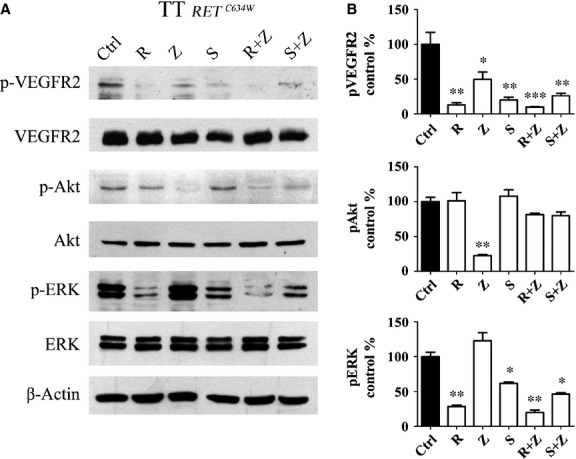
Western blot analysis. Representative Western blot of β-actin, ERK, phospho-ERK, Akt, phospho-Akt, VEGFR2, phospho-VEGFR2 antibodies in TT cells treated for 4 hrs using IC50 doses. (A) Ctrl, untreated cells; R, cells treated with RAF265; Z, cells treated with ZSTK474; S, cells treated with SB590885; R+Z, cells treated with RAF265+ZSTK474; S+Z, cells treated with SB590885+ZSTK474. These profiles are representative of 3 separate experiments. (B) Quantitative densitometry is shown to compare phosphorylated protein expression as a ratio normalized to untreated control levels. One-way anova: *, *P* < 0.05; **, *P* < 0.01; ***, *P* < 0.001.

To show the effects of the BRAF and PI3K inhibitors on their targets, we used Western blot to test the drugs both separately and in combination on the phosphorylation of ERK and Akt (Fig.4A and B). At basal level the TT cells expressed similar amounts of total Akt and total ERK. RAF265, alone and in combination with ZSTK474, strongly inhibited ERK phosphorylation (*P* < 0.01), while SB590885, alone and in combination with ZSTK474, induced a smaller but still significant reduction in ERK phosphorylation (*P* < 0.05). Only ZSTK474 alone prompted a significant reduction in the phosphorylation signal in the Akt signalling pathway compared to the control (*P* < 0.01).

As RAF265 also inhibits the VEGFR2 17, we evaluated the expression of this receptor in TT cells (Fig.4A and B). At basal level, the cells expressed similar amounts of total VEGFR2 with all the treatments. We found phosphorylated VEGFR2 strongly expressed in the untreated cells, while markedly reduced reactivity was apparent on the phosphorylated receptor after RAF265 treatment (*P* < 0.01) and this effect was enhanced when the RAF265+ ZSTK474 combination was tested (*P* < 0.001).

### Cell cycle distribution

**Figure 5:**
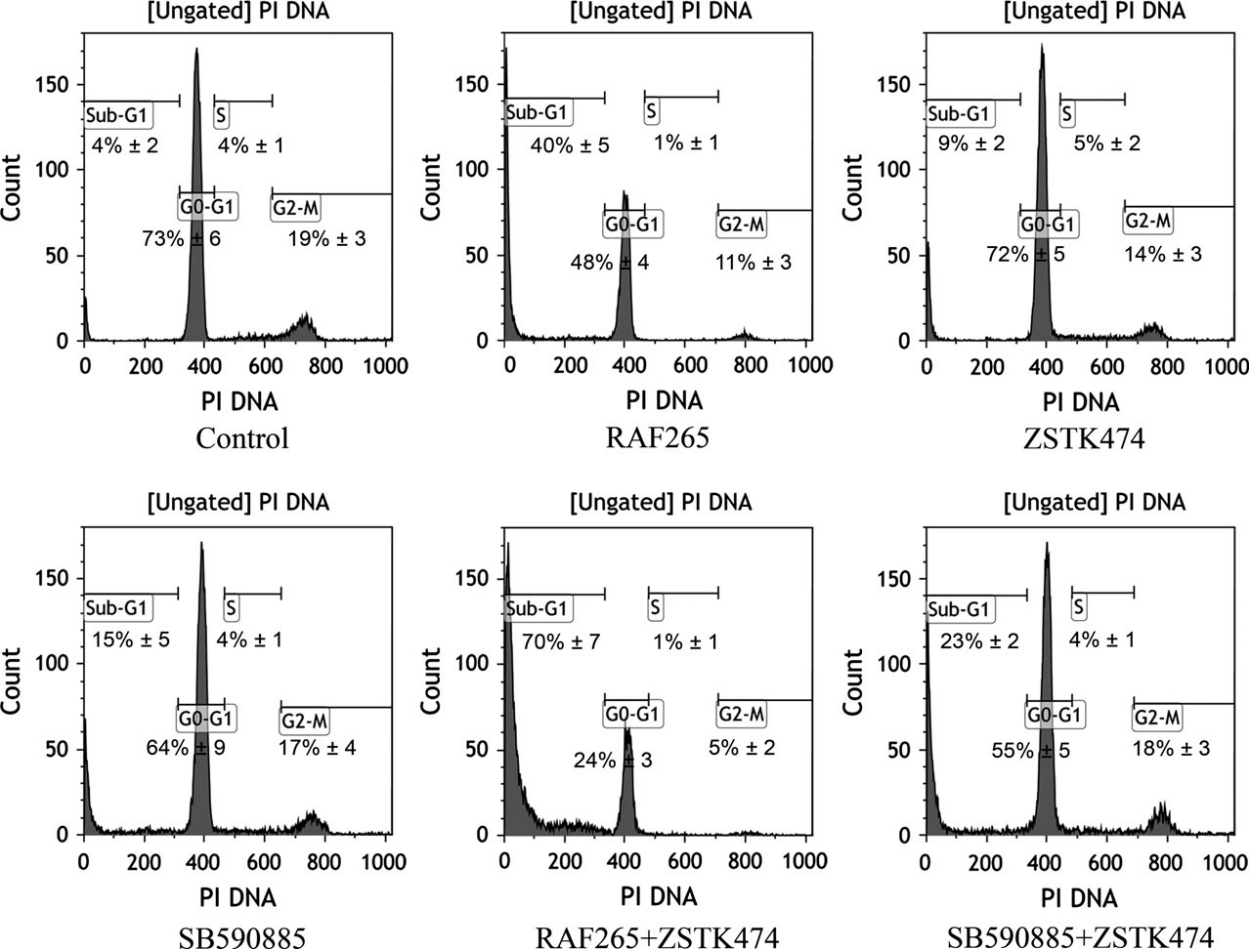
Cell cycle analysis following drug treatments. Representative cell cycle distribution analysis at 72 hrs for TT cells left untreated or treated with drugs using IC50 doses. Four cell phases (G2/M, S, G0-G1, sub-G1) with their percentages alongside are indicated in each plot. Representative distribution of cell cycle analysis, performed in triplicate.

We analysed the effects of the drugs on TT cell cycle distribution (Fig.5). RAF265 was the most effective single drug, inducing a substantial increase in the sub-G1 phase from 4% in untreated cells to 40% (*P* < 0.01), with a concomitant G0-G1 phase reduction (from 73% in untreated cells to 48% in treated cells). ZSTK474 and SB590885 each induced a slight but significant increase in the sub-G1 phase (*P* < 0.05). The combination of RAF265+ ZSTK474 was highly effective in inducing a remarkable increase in the sub-G1 phase, from 4% in untreated cells to 70% (*P* < 0.001), with a concomitant decrease in the G0-G1 phase. The combination of SB590885+ ZSTK474 induced a moderate increase in the sub-G1 phase, from 4% in untreated cells to 23% (*P* < 0.01), and a concomitant reduction in the G0-G1 phase.

### Apoptosis

**Figure 6:**
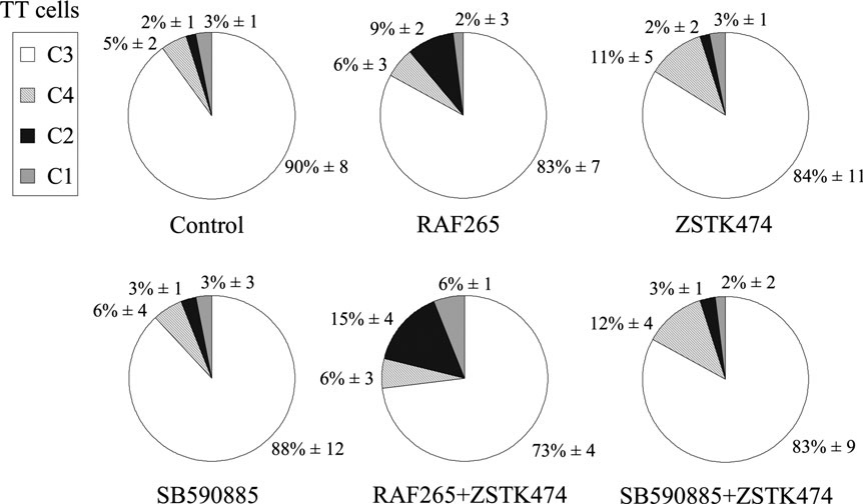
Apoptosis analysis following drug treatments. Representative flow cytometry after annexin V-FITC/propidium iodide staining in TT cells at 72 hrs. Cells were treated with all drugs, alone or in combination, using IC50 doses. Pie graphs indicate the percentages of C1 (dead cells), C2 (necrosis), C3 (live cells), C4 (early apoptosis). Results are representative of three separate experiments.

Flow cytometry after annexin V-FITC/propidium iodide staining was performed to discriminate between apoptosis and necrosis (Fig.6). The results are shown in a specific flow cytometry diagram, where C3 indicates live cells, C1 dead cells, C4 early apoptosis and C2 necrosis. A sustained increase in necrotic TT cells was apparent after treatment with RAF265, alone and in combination with ZSTK474 (from 2% in untreated cells to 9% in cells treated with RAF265, and 15% in those treated with both RAF265 and ZSTK474, *P* < 0.05). A significant increase in early apoptosis, compared with the control, was also seen for ZSTK474, both alone (from 5% to 11%, *P* < 0.05) and in combination with SB590885 (from 5% to 12%, *P* < 0.05).

### Effects of drugs on CT gene expression and secretion in TT cell lines

**Figure 7:**
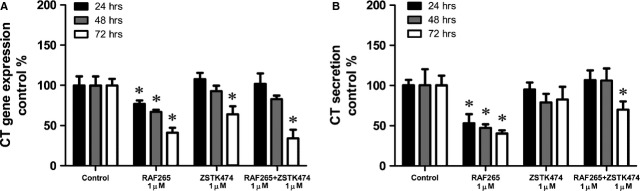
Drug effects on TT calcitonin production. Effect of RAF265 and ZSTK474, alone and in combination, on calcitonin mRNA abundance and secretion, in TT cells. (A) Calcitonin mRNA expression levels in treated cells, compared with the control, as measured by quantitative PCR at 24, 48 and 72 hrs. (B) Calcitonin secretion in TT cells in conditioned medium, compared with the control, as measured by LIAISON® Calcitonin_II-Gen at 24, 48 and 72 hrs. One-way anova: *, *P* < 0.05.

We examined the effects of the drugs on CT gene expression *in vitro*. As shown in Figure7A, treating the TT cells with RAF265 alone caused a significant drop in CT mRNA expression, which was especially evident after 72 hrs. ZSTK474, alone and in combination with RAF265, took 72 hrs to induce a significant decrease in CT mRNA expression level. Treatment with SB590885, alone or combined with ZSTK474, had no effect on CT mRNA levels.

Then we examined the effects of the drugs on CT levels in the TT cells in the culture medium by chemiluminescent immunoassay. As shown in Figure7B, the CT levels dropped rapidly and were significantly lower than in the control after treatment with RAF265 alone for different times (24, 48 and 72 hrs), and after treatment with RAF265+ ZSTK474 for 72 hrs. Treatment with SB590885, alone or in combination with ZSTK474, demonstrated no clear effect on CT secretion (data no shown).

## Discussion

There is currently no effective systemic treatment for patients with metastatic MTC, conventional chemotherapy and/or radiotherapy having demonstrated only a limited efficacy in inducing tumour remission or stabilization 18,19. New approaches with tyrosine kinase inhibitors have been found to prolong progressive disease-free survival in advanced MTC patients, but they demand long-term treatments that have non-negligible adverse effects 20. Hence the urgent need for new therapeutic modalities.

The activated RET kinase is known to signal *via* Ras/Raf/MEK/ERK and PI3K/Akt pathways, among others, leading to enhanced proliferation and resistance to apoptosis 11. Simultaneously inhibiting MAPK and PI3K could have a therapeutic effect in patients with oncological diseases carrying these signalling pathways in a constitutively active state.

The present work aimed to examine the effects of three new drugs, RAF265, SB590885 and ZSTK474, on a RET-activated TT cell line model derived from an aggressive medullary thyroid carcinoma. TT cells retains some differentiated properties of the normal thyroid C cells such as CT production and are one of the most representative models for the study of MTC. RAF265 is a BRAF inhibitor compound specific for the V600E-mutated isoforms that has been designed and developed primarily for the treatment of locally advanced and metastatic BRAF-mutated melanoma. A phase 2 clinical trial has just been completed (ClinicalTrials.gov identifier NCT00304525), the results of which have yet to be published, but over the years several studies have demonstrated an effect of this compound in other tumours, including differentiated thyroid cancers. A previous publication by our group showed that RAF265 had an inhibitory effect on papillary thyroid cancer (PTC) cell lines harbouring the BRAFV600E mutation 14. RAF265 alone has also already been tested in TT cells, and found to induce a significant decrease in TT cell viability 21. The present work is the first, to the best of our knowledge, to examine MAPK pathway inhibition by either RAF265 or SB590885 (the latter compound tested here for the first time) in combination with PI3K pathway inhibition by ZSTK474. We did not use RAF265 and SB590885 together because they compete for the same ATP-binding site of the RAF dimer, and this overlap may limit the effectiveness of the two drugs.

The most evident effects on TT cell viability were obtained by RAF265, both alone (with an IC50 of 0.09 μM) and in combination with ZSTK474 (with a median CI of around 0.22). SB590885 was also effective in reducing TT cell viability on its own, while combining it with ZSTK474 only showed an additive effect.

A previous report suggested that the effect of a mutated BRAF inhibitor, RAF265, on a medullary cell line like TT, carrying a wild-type BRAF but harbouring a RETC634W mutation, could be explained by an inhibitory effect on the activated RET isoforms 21. In our work, we used Western blot analysis to try and see if other cellular pathways could be repressed after the RAF265 treatment. In TT cells, the p-ERK signal was active at the baseline and both BRAF inhibitors were able to inhibit it, but RAF265 (alone or in combination with ZSTK474) had the strongest effect, not excluding a direct influence on the wild-type BRAF isoforms. RAF265 also exerted a direct inhibitory effect on activated VEGFR2: in our cells, the phosphorylated VEGFR2 signal (constitutively triggered at basal level) was inhibited by RAF265 alone and even more so when it was combined with ZSTK474.

When we analysed the effects on cell cycle distribution, we observed a general increase in the sub-G1 fraction of cells treated with SB590885+ ZSTK474, and especially with the RAF265+ ZSTK474 combination, with no concomitant increase in the G0-G1 phase. These results were unexpected and inconsistent with our previous findings in a PTC model, and with the results obtained with other kinase inhibitors on differentiated thyroid cancer cell lines. In the work by Salerno *et al*., for instance, PLX4032 and PLX4720 (two BRAF V600E ATP-competitive kinase inhibitors) inhibited G0-G1 and altered the expression of genes involved in the control from the G1 to the S phase 22. The Authors used several thyroid cancer cell lines, but not MTC, so the effect on the cell cycle in the TT line might be linked to the specific inhibition of the mutated RET protein.

With the aid of annexin V-FITC/propidium iodide staining, we tried to elucidate the cell death mechanism underlying the sub-G1 fraction increase: we found a necrosis-like mechanism with no significant increase in the apoptotic cell fraction, especially after treatment with the RAF265+ ZSTK474 combination. The effect of RAF265, alone and with ZSTK474, appeared to be cytotoxic and not cytostatic. In our opinion, this last finding seems to be particularly relevant for the purpose of designing future therapies because the main tyrosine kinase inhibitors currently used in clinical practice have only revealed cytostatic effects and need to be administered non-stop in MTC patients 20.

In short, RAF265 has a direct inhibitory effect on upstream signalling pathway receptors (mutated RET and activated VEGFR2), but also on the BRAF intermediate MAPK pathway effector. This action, combined with the inhibitory effect of ZSTK474 on the p-AKT pathway, can explain the synergistic effect observed on TT cell line (Fig.1).

Rearranged during transfection kinase mediates a physiological pathway that controls CT secretion. Akeno-Stuart *et al*. have shown that NVP-AST487 (a *N*,*N*′-diphenyl urea with an IC50 of 0.88 μM on RET kinase) inhibits CT production in TT cells *in vitro*, also limiting gene transcription 23. We quantified CT mRNA and protein production and found the same effect in TT cells after RAF265 treatment, which goes to show that RAF265 has a powerful inhibitory effect on the CT production pathway, controlled by RET.

This study provides the first demonstration of the strong inhibitory effects of a novel BRAF inhibitor, RAF265, and its synergistic effect with the PI3K inhibitor ZSTK474, in TT cells harbouring genetic alterations in RET. More preclinical research on MTC has focused on RET inhibitors, but to date no studies had examined the possible effects of RAF inhibitors in MTC. Given the extremely common genetic alterations in the particularly aggressive and currently untreatable MTC, combination therapy with RAF265 and ZSTK47 may prove effective for this aggressive cancer. As the two inhibitors have been successfully tested separately in clinical trials on other human cancers, the preclinical findings reported in this study warrant clinical trials on their combined use for treating aggressive MTC.
